# A one month high fat diet disrupts the gut microbiome and integrity of the colon inducing adiposity and behavioral despair in male Sprague Dawley rats

**DOI:** 10.1016/j.heliyon.2022.e11194

**Published:** 2022-10-27

**Authors:** Gladys Chompre, Lubriel Sambolin, Myrella L. Cruz, Rafael Sanchez, Yarelis Rodriguez, Ronald E. Rodríguez-Santiago, Yasuhiro Yamamura, Caroline B. Appleyard

**Affiliations:** a Biology and Biotechnology Department, Pontifical Catholic University of Puerto Rico, Ponce, Puerto Rico; b Basic Sciences Department, Division of Physiology, Ponce Health Sciences University/Ponce Research Institute, Ponce, Puerto Rico; c Basic Sciences Department, Division of Pharmacology, Ponce Health Sciences University/Ponce Research Institute, Ponce, Puerto Rico; d AIDS Research Infrastructure Program, Ponce Health Sciences University/Ponce Research Institute, Ponce, Puerto Rico

**Keywords:** Gut microbiome, Gut integrity, Claudin-5, Bacteriodes, Tight junction proteins, S24-7, LPS, Microglia, Phospho-mTOR, High Fat Diet

## Abstract

High-fat diet (HFD) is associated with gut microbiome dysfunction and mental disorders. However, the time-dependence as to when this occurs is unclear. We hypothesized that a short-term HFD causes colonic tissue integrity changes resulting in behavioral changes. Rats were fed HFD or low-fat diet (LFD) for a month and gut microbiome, colon, and behavior were evaluated. Behavioral despair was found in the HFD group. Although obesity was absent, the HFD group showed increased percent weight gain, epididymal fat tissue, and leptin expression. Moreover, the HFD group had increased colonic damage, decreased expression of the tight junction proteins, and higher lipopolysaccharides (LPS) in serum. Metagenomic analysis revealed that the HFD group had more Bacteroides and less S24-7 which correlated with the decreased claudin-5. Finally, HFD group showed an increase of microglia percent area, increased astrocytic projections, and decreased phospho-mTOR. In conclusion, HFD consumption in a short period is still sufficient to disrupt gut integrity resulting in LPS infiltration, alterations in the brain, and behavioral despair even in the absence of obesity.

## Teaser

Gut microbiome; Gut integrity; Claudin-5; Bacteriodes; High fat diet; Tight junction proteins; S24-7; LPS; Microglia; Phospho-mTOR.

## 1 Introduction

The prevalence of obesity has increased over the last few decades in all age groups worldwide (Faith et al., 2011; Ng et al., 2014). Recent statistics from the Center for Diseases Control and Prevention show that around seventy percent of adults of twenty years old and over are overweight (Fryar et al., 2020). A high fat diet (HFD) is related to increased body mass index and associated with obesity; however, body weight does not necessarily correlate with the amount of adiposity in the body or metabolic disorders (Malik et al., 2013; Oliveros et al., 2014). Regardless, a HFD has been associated with comorbidities such as hypertension, atherosclerosis, diabetes, metabolic syndrome, and depression (Laborde and Sáez-Santiago, 2013; Goodpaster et al., 2005; Ford, 2004; Stunkard et al., 2003). Importantly, more than 10 million American adults suffer from mood disorders including major depressive disorder, dysthymic disorder, and bipolar disorder according to the U.S. Census Bureau Population Estimates by Demographic Characteristics (Kessler et al., 2005; Baxter et al., 2014). Although several studies in both obese humans and animals have found depressive-like behavior (Stunkard et al., 2003; Baxter et al., 2014; Reeves et al., 2008; Willner et al., 1992; Gotlib and Joormann., 2010), and lately there is increased awareness of the importance of gut-brain interactions on behavior, it is still not clear how alterations in the gut microbiome due to dietary habits might be associated with behavioral changes.

Prior studies reported that the human gut microbiome has approximately one trillion bacteria including one thousand species, but recent studies using culturomics used to understand the bacterial diversity to species reveal that the gut microbiome genome has higher than the human genome (Methé et al., 2012; Lagier et al., 2018; Almeida et al., 2019). Next generation sequencing methods have helped to ascertain the bacterial composition of different fluids, including the feces, using 16S ribosomal RNA (Qin et al., 2010). According to the Human Microbiome Project Consortium, healthy gut human samples are dominated by two phyla: *Firmicutes* and *Bacteroidetes*, and it is known that the microbiome changes with various diseases including obesity (Huttenhower and Human Microbiome Project Consortium, 2012; Gregor and Hotamisligil., 2011; Clemente et al., 2012). It has been demonstrated that gut microbiome from obese rodents transplanted into lean rodents changes several characteristics of the recipient animals including fat accumulation, gut inflammation, and behavior, to resemble that of the donor obese rodent (Baxter et al., 2014; Considine et al., 1996; Bäckhed et al., 2007; Bruce-Keller et al., 2015). Increased levels of *Bacteroidetes, Actinobacteria,* and *Proteobacteria* are present in patients with depression (Jiang et al., 2015), which also correlates strongly with *Alistepes* and *Oscillibacter* (Naseribafrouei et al., 2014), and these can be associated with inflammatory pathways that may be linked to gut permeability. Importantly, permeability in the gut is normally tightly controlled to ensure that the appropriate defense mechanisms occur, while at the same time allowing absorption of the necessary nutrients. An imbalanced diet, such as that containing high levels of fat, has been associated with decreased gut motility, decreased tight junctions, and increased gut permeability (Kim et al., 2012; Cani et al., 2008). Consumption of a high fat, low carbohydrate diet in mice for 4 weeks decreased tight junctions allowing the translocation of bacterial products such as lipopolysaccharide (LPS) from the gut to the blood, activating the immune system to increase inflammation (Cani et al., 2008).

Prior studies have found that consumption of an HFD for more than 10 weeks induces obesity and insulin resistance, and is related to behavioral changes such as impaired cognition, anxiety, and depressive-like behavior (Bose et al., 2008; Cordner and Tamashiro, 2015; Calligaris et al., 2013; Privitera et al., 2011). Additional studies found that long-term consumption of HFD also causes neuroinflammation (Miller and Spencer., 2014; Almeida-Suhett et al., 2017). Interestingly, long-term but not short-term exposure to the microbiome from HFD-fed mice is associated with insulin resistance (Foley et al., 2018). The impact of a shorter length HFD is unclear: in one study Sprague-Dawley rats 7 days on an HFD appeared to have anti-depressive like effects with decreased immobility in a forced swim test (Sumaya et al., 2016), while mice on an HFD of 8 weeks developed depressive-like behavior only when concomitant administration of low levels of corticosterone occurred (Liu et al., 2014). In short, the link between an HFD over a short-term period, the gut microbiome, LPS, and changes in behavior is still not well understood. We hypothesized that a short-term HFD can cause changes in the microbiome, and in the integrity of the colonic tissue, resulting in behavioral changes. The specific research objectives of this study were to evaluate whether an HFD given over a short period of time can alter the gut microbiome and the structural integrity of the colonic tissue; and then to assess if these changes resulted in translocation of bacterial products and systemic inflammation, which would correlate with behavioral changes.

## 2 Experimental model and subject details

Two-month-old male Sprague Dawley rats were purchased from the Ponce Health Sciences University Animal House having a diet of Prolab® Rat/Mouse/Hamster 3000. This age represents young adults equivalent to approximately eighteen human years which were subjected to a diet intervation of 30 days which represents around 2 human years (Sengupta et al. 2012, 2013; Quinn et al. 2005). For the dietary intervention they were fed either a Low-Fat Diet (LFD; 10% fat, 20% protein and 70% carbohydrate, Research Diet, New Brunswick NJ Cat. No. D12450J, n = 14) or High-Fat Diet (HFD; 60% fat, 20% protein and 20% carbohydrate, Research Diet, New Brunswick NJ Cat. No. D12492, n = 14) for a period of one month (Table 1, and supplemental data). These diets were selected based on studies by Novak et al. who demonstrated that a similar HFD increased weight and changed spontaneous physical behavior (Novak et al., 2006), and Dutheil et al., 2016 who showed behavioral changes between a LFD and HFD using a longer 60 day time period. Animals were kept in a bio-bubble on a twelve-hour light/dark schedule being individually housed with sterile bedding. Food and water were provided *ad libitum* and animal weights recorded every day during handling. Obesity in animal models can be defined based on animal weight and/or increased body fat content, with an increase of 10-25% body weight over normal chow fed age-matched rats commonly reported as moderate obesity, and >40% as being severe (Hariri and Thibault, 2020). Every week, the cages were cleaned with alcohol and sterile bedding was replaced. Thirty days after commencing the diet, animals underwent behavioral testing using open field and forced swim tests. All procedures were approved by Ponce Health Sciences University Institutional Animal Care and Use Committee protocol #199 and carried out in accordance with the National Institutes of Health guide for the care and use of laboratory animals.

## 3 Method details

### 3.1 Open field test

An open field test was performed as previously described (Ramos-Ortolaza et al., 2017). The open field arena consisted of a square wood box covered with black Formica (W 36 x L 36 x H 18 inches), located in a small isolated room (7.5 x 7.5 feet) with dim red light. For the test, animals were placed in the center of the arena and allowed to roam for 10 minutes. A video camera was placed above the arena to record the animals during the test. The ANY-maze software (Stoelting Co., IL.) was used to track time spent at the central and peripheral zones of the arena, total distance traveled and velocity, as measures of anxiety-like behavior (Prutt et al., 2003; Gould et al., 2009). White noise contained in the ANY-maze software were used to reduce variations in environmental sounds. Fecal pellets were also counted as an indirect measure of anxiety-like behavior (Malliots et al., 2000; Cuevas et al., 2012). The arena was cleaned with 100% ethanol between animals to remove scent cues that could potentially affect their behavior.

### 3.2 Forced swim test

Immediately after the open field test, a Forced Swim Test (FST) was performed to measure behavioral despair as previously described (Ramos-Ortolaza et al., 2017). Briefly, animals were placed in a glass cylinder (40cm in height and 30cm in diameter) filled with water (30± 1°C) to a height of 20cm with 15cm above the head of the rat. This level was high enough to avoid the animals touching the bottom with their tails, but far enough below the top edge of the cylinder to prevent them from being able to escape. The trial was recorded for 10 minutes. ANY-maze software was used to manually track immobility, swimming, struggling and diving. In addition, white noise was used to avoid the impact of the environmental sound variation. Immobility was measured when the rats were floating or slightly moving their forepaws to keep their nose above the water; swimming was measured when the rats were moving horizontally while keeping the nose above the water; struggling was measured when the rats were moving their forepaws rapidly and breaking the surface of the water; and diving was measured when the rats completely submerged to the bottom of the cylinder. Escaping, touching the bottom of the cylinder with the tail, or giving up and sinking was used as criteria to exclude animals from the analysis. Two animals from the LFD and one from the HFD group were removed under these exclusion criteria. At the end of the FST, animals were removed from the cylinder and dried under warm light for 10 minutes. Only one FST session was performed, with no acclimation period, to prevent confounding effects of learning (Wulsin et al., 2010; De Pablo et al., 1991).

### 3.3 Euthanasia and sample collection

Twenty-four hours after behavioral testing, rats were deeply anesthetized with pentobarbital (45 mg/kg i.p.). A cardiac puncture was used to obtain blood after verifying non-responsiveness. A laparotomy was performed and epididymal fat pads were removed carefully, weighed and snap frozen on dry ice. The distal colon was removed and opened longitudinally to allow the collection of feces and macroscopic examination. Feces were transferred to labeled tubes and snap frozen on dry ice. One half of the colon was formalin fixed and processed for H&E staining and the other was removed and snap frozen on dry ice for molecular analysis. The brain was removed and mid brain sections were formalin fixed for staining (see sections below).

### 3.4 Colonic damage and crypt length

The colon specimens were analyzed in a blinded fashion for the presence of diarrhea, ulceration, thickness (mm), and adhesions to give a total macroscopic damage score (Appleyard and Wallace, 1995). Using previously described procedures H&E stained sections were examined for histological changes by two different observers using the following criteria: loss of mucosal architecture (from 0 to 3: absent, to severe), muscle thickness (from 0 to 3: zero meaning 1/2mucosal thickness, one meaning ½ to ¾ mucusal thickness, two meaning equal to mucosal thickness, and three meaning all muscles) , cell inflitration (zero non infiltration, one in the muscularis mucosae, two *in lamina propria/villi* , and three *in serosa*), crypt abscess formation (present (1) or absent (0)), and goblet cell depletion (present(1) or absent (0)), were evaluated (Appleyard and Wallace, 1995, Hernandez et al., 2003). Photos were taken under the 40x objective. Separately, images were examined and the crypt length measured using Image J computer software from the National Institutes of Health. Images were examined using three similar fields and three measurements of crypt length (from the bottom to top of the crypt) were obtained and averaged.

### 3.5 Claudin-5 and Occludin mRNA expression

Thirty milligrams of snap frozen colonic tissue were transferred to mRNAse free microcentrifuge tubes filled with beads and homogenizing solution from the AllPrep DNA/RNA/Protein Minikit from Qiagen Co. (Cat. No. 80004). Tubes were transferred to the Bullet blender from Advance Co and homogenized for five minutes before extracting RNA following the Qiagen manufacturer’s procedure measuring the concentration and quality of the RNA using nanodrop 2000 (Thermoforma Co). One microgram of mRNA was changed to complementary DNA using iScript cDNA synthesis kit from Biorad Co. (1708891). Realtime Polymerase reaction was performed using iQ SYBR Green Supermix (1708882) from Biorad Co. and primers from Qiagen Co (Claudin-5 PPR46476A; Occludin PPR48441A, with beta actin PPR06570C as internal control). Data was reported as a fold change using the equation 2^-δCT^ and normalized by the control (LFD).

### 3.6 Fecal metagenomics

DNA purified from rat feces pellets was used as template for PCR targeting the V1-V3 region of the bacterial 16s rDNA gene for the microbiome analysis. Amplification was verified by gel electrophoresis and successfully amplified samples were then quantified using the Qubit 2.0 (Life Technologies). MiSeq libraries were prepared using the Nextera XT DNA kit (Illumina LLC), according to manufacturer protocol. The final libraries were loaded in a MiSeq instrument with a 500 cycles kit.

MiSeq data was extracted, decompressed, and analyzed using the QIIME software (v.1.9.0) in a Linux platform (Ubuntu). Forward and reverse reads (FASTQ file) of each sample were joined using the join_paired_ends.py script with QIIME default parameters. The resulting reads were split libraries and then filtered by quality and length, reads shorter than 12 bp and with a quality less than Q30 were discarded. A closed OTUs was picked using the greengenes 13_8-release database with a 97-similarity threshold. The generated OTU table was rarefied to an equal number of OTUs per sample using the single_rarefication.py QIIME command ad used for downstream analyses and other statistical data. Raw data is available at National Center for Biotechnology information (NCBI) with accession number: PRJNA870914.

### 3.7 Immunofluorescence assay

IBA-1 and GFAP immunofluorescence staining was performed in previously preserved tissue embedded in paraffin. After sacrifice, brain was extracted and fixed in 10% paraformaldehyde. Tissue sections were embedded in paraffin and coronal sections of dorsal were cut at 8 μm using a microtome and placed on a positively charged slide. Slides were deparaffinized in xylene followed by hydration in descending grade of ethanol (100% two times, 95%, 80%, and 70%, CDA-19), for 3 minutes each. Tissues were washed for 1 minute with distilled water and placed in PBS solution for 5 minutes prior to the antigen retrieval incubation made with 0.01 M Citrate-EDTA buffer (pH = 6.2) at 90–95 °C for 40 min. Tissues were washed two times for 2 minutes with distilled water and were placed in PBS for 5 minutes. Then, protein block (Cat No. 50062Z, Life Technologies, Frederick, MD) step was performed for 15 minutes to avoid non-specific bindings on the tissue. Tissues were incubated with Anti-Iba-1 rabbit polyclonal primary antibody (Cat no. 019-19741, FUJIFILM Wako Pure Chemical Corporation) or Anti-GFAP mouse monoclonal primary antibody (Cat no. 644702, BioLegend) overnight in a humidified chamber at 4 °C. The day after, slides were washed twice with PBS for 5 minutes and incubated during 5 minutes with Goat Anti-Rabbit IgG Secondary Antibody conjugated with Alexa Fluor 555 (Cat no. A21429, Invitrogen by Thermo Fisher Scientific) for the Anti-Iba-1 stained slices and Goat Anti-Mouse IgG Highly Cross-Adsorbed Secondary Antibody, Alexa Fluor 488 (Cat no. A11029, Invitrogen by Thermo Fisher Scientific) for Anti-GFAP stained slices during 30 min at room temperature. Then, slices were washed two times by PBS for 5 minutes each, followed by incubation with DAPI during 5 minutes for cell nuclei labeling. Tissues were washed twice with PBS for 5 minutes and coverslip were mounted on the slides with ProLong Gold antifade (Cat No. P36934, Invitrogen by Thermo Fisher Scientific). Samples were taken using an Olympus System Microscope Model BX60 (Olympus Life Sciences Solution). ImageJ software was used to measure intensity, percent of area for microglia and length for GFAP stainig(for each field, lenght and number of projection were measure) . The values are reported as mean ± SEM.

#### 3.7.1 Phospho m-TOR Immunohistochemistry

Brain sections were cut at 4μm thickness with a microtome (Microm HM340, Microm International) and mounted on glass slides. Tissue sections were deparaffinized with xylene, 2 changes, 15 minutes each, and hydrated in descending grades of ethanol to distilled water. This was followed by a 3% Hydrogen peroxide (Sigma-Aldrich) incubation for 15 minutes to block endogenous peroxidase and a fine minute PBS wash. After antigen retrieval (0.01M Citrate-EDTA buffer, pH 6.0, 95-99°C for 40 minutes), slides were left for 20 minutes at room temperature, rinsed with 2 changes of distilled water for 2 minutes and placed in PBS for 5 minutes. Slides were blocked with normal goat serum (BioGenex, cat#HK112-9KE) for 15minutes and followed by an overnight incubation with primary antibody (Phospho- mTOR antibody, cat# 2976, Cell signaling; 1/10 dil.). A negative control with PBS instead of primary antibody was run in each slide. On the second day, slides were washed with PBS for 5 minutes. A Multi Link was used as the secondary antibody for 20 minutes, followed by PBS wash for 5 minutes. The slides were placed in the Streptavidin Peroxidase for 20 minutes (Super Sensitive Link-Label IHC Detection System, cat#LP000-UCLE, BioGenex, San Ramon, CA, USA). For development, one drop of 3,3’ Diaminobencidine (DAB) (cat# HK153-5KE, BioGenex, San Ramon, CA, USA) was used on each tissue and the exposure was monitored for 45 seconds under a light microscope. Then, the slides were washed with running water for 5 minutes, dehydrated through graded alcohol, cleared with xylene and mounted with Cytoseal XYL (cat# 8312-4, Richard Allan Scientific, Kalamazoo, MI, USA). After representative areas were photographed at high power field for each slide, the intensity and percent of area was determined.

### 3.8 Cytokine bead array and lipopolysaccharide

After removing the red blood cells by centrifugation, the serum was transferred to a sterile tube and stored at -20°C. Serum was analyzed using bead array following the manufacturer’s procedure (Millipore, Billerica, MA; Cat. No HBNMAG-51K) and Kinetic QCL Chromogenic Assay (Millipore, Billerica, MA; Cat. No NC9597521) to measure LPS.

## 4 Quantification and statistical analysis

### 4.1 Statistical analyses

Prior to carrying out the study a power analysis was performed by the epidemiology department at Ponce Health Sciences University using Epidat software version 3.1 as required by the IACUC. Animals were assigned a number then distributed randomly with blinded analysis to avoid bias in scoring. Data were reported as the average and standard error of the means. A student t-test was used for absolute weight or percent weight change, absolute weight per day with repeated student t-test measures analysis. A student’s T-test was used to analyze significance for the overall data, Mann-Whitney test was used for microbial data such bacterial abundance, and Spearman correlation was used. All were reported as p < 0.05(*), p < 0.01(**), and p < 0.001(***). For metagenomics: for the alpha-diversity, measurements were calculated and analyzed by a non-parametric student t-test and using 999 Monte Carlo permutation, Beta-diversity using ANOSIM test, Bacterial abundance was analyzed through non-parametric student t-test with bonferroni post hoc. All analyses were done on GraphPad version 7.0. ANOSIM test was done with Qiime 1.9.1(41).

## 5 Results

### 5.1 Thirty days of HFD feeding results in higher percent weight gain and epididymal fat tissue than LFD

**Figure 1:**
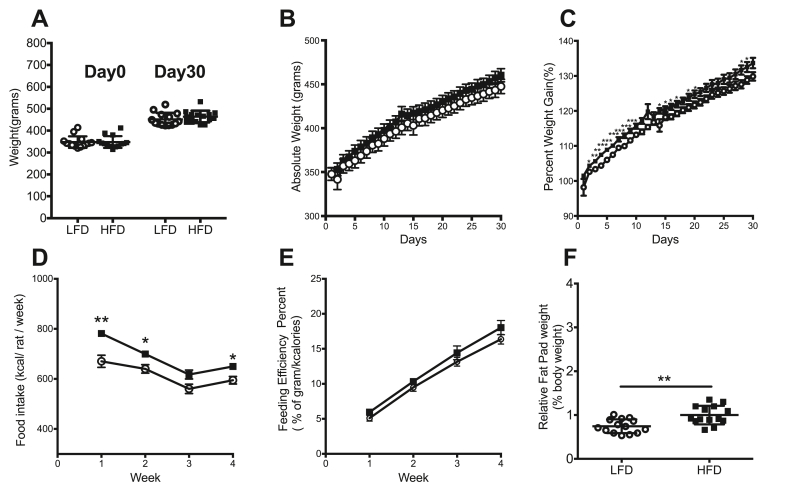
Thirty days of HFD feeding results in higher percent weight gain and epididymal fat tissue than LFD. Before switching to either the HFD or the LFD, the absolute weight (A) of the LFD group and the HFD group were similar (day 0). No significant differences were observed during the dietary intervention, or at time of sacrifice (day 30) between animals on the LFD and HFD (B). However, (C) animals in the HFD group showed a larger increase in percent weight gained at several time points during the intervention. (D) Animals fed with HFD consumed more kilocalories than LFD during week 1, week 2 and week 4 (n = 6/group). (E) Both groups showed no significant differences in feeding efficiency percent during the four weeks (n = 6/group). (F) Additionally, the epididymal fat pads were found to be significantly heavier in the HFD group compared to LFD. Data represented as mean ± standard error of the mean; n = 14/group except where indicated otherwise. ∗p < 0.05, ∗∗p < 0.01, ∗∗∗p < 0.001.

Before switching to either the HFD or the LFD, the absolute weight of the LFD group (349.4 ± 9.02 g) and the HFD group (349.4 ± 9.18 g) were similar (Figure 1A, n = 14/group). After the 30-day dietary intervention animals were weighed and again no significant differences were found between the LFD (452.8 ± 9.37 g) and the HFD (467.9 ± 9.35 g) with both groups gaining weight (Figure 1A, n = 14/group**)**, nor in the absolute weight per day during the month (Figure 1B, n = 14/group**)**. We also checked to see if animals were gaining weight taking into consideration their weight at day zero. Animals in the HFD group showed a larger increase in percent weight gained on several days during the protocol (days 2, 3, 4, 5, 6, 7, 8, 9, 10, 11, 14, 16, 17, 20, 28, 29) compared with the LFD group (p < 0.05; Figure 1C, n = 14/group). As expected, animals fed with HFD consumed more kilocalories per animal per week than LFD during week 1 (781.5 ± 10.85 kcal/rat/week vs 670.4 ± 24.32 kcal/rat/week), week 2 (617.4 ± 17.88 kcal/rat/week vs. 560.3 ± 18.76 kcal/rat/week), and week 4 (649.5 ± 12.30 kcal/rat/week vs. 594.7 ± 15.30 kcal/rat/week; p < 0.05 (Figure 1D, n = 6/group). Moreover, there was no difference between the groups in feeding efficiency during week 1 (5.1 ± 0.42 % vs. 6.0 ± 0.32%), week 2 (9.5 ± 0.56% vs. 10.3 ± 0.42%), week 3 (13.2 ± 0.63% vs. 14.4 ± 0.99%), or week 4 (16.4 ± 0.70% vs. 18.0 ± 1.02%); p > 0.05 (Figure 1E, n = 6/group). Additionally, the relative epididymal fat pad percent was found to be significantly higher in the HFD group (1.0 ± 0.06%) compared to LFD (0.8 ± 0.04%; p < 0.01; Figure 1F, n = 14/group).

### 5.2 Animals on HFD show behavioral despair in the forced swim test

**Figure 2:**
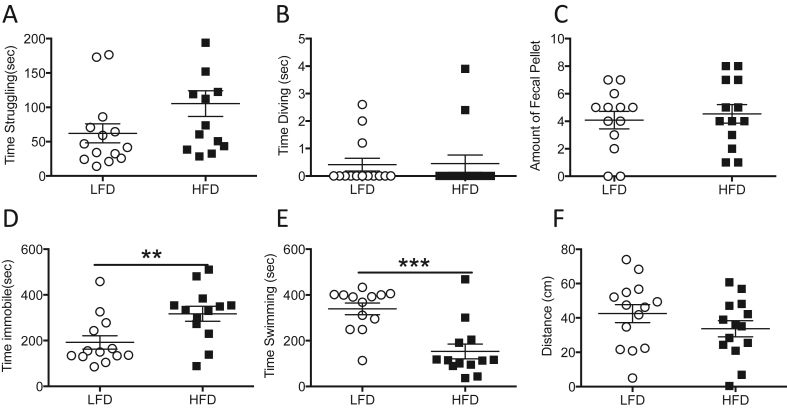
**Animals on HFD show behavioral despair in the forced swim test**. Two-month-old male Sprague Dawley rats fed with LFD or HFD showed no significant differences in (A) time spent struggling, or (B) time spent diving during the Forced Swim Test, and no significant differences in (C) fecal pellet output. However, rats receiving the HFD spent (D) more time immobile, (E) less time swimming, and (F) covered less distance. Data represented as mean ± standard error of the mean. n = 14 in LFD group and n = 14 in HFD group. ∗p < 0.05, ∗∗p < 0.01.

After a month on either the HFD or the LFD, the behavior of the rats was assessed in the forced swim test. An increase in immobility time, or a decrease in swimming or struggling in the forced swim test can indicate despair behavior in rodents. Our analysis found no statistically significant differences in struggling (Figure 2A, n = 14/group, LFD, 62.11 ± 13.90 s; HFD, 105.4 ± 19.0 s, p > 0.05) or diving (Figure 2B, n = 14/group, LFD, 0.4 ± 0.23 s; HFD, 0.45 ± 0.32 s, p > 0.05). No significant differences were found in fecal pellet count either (Figure 2C, n = 14/group, LFD, 4.1 ± 0.64 FPC vs HFD, 4.6 ± .067 FPC). However, the HFD group were more immobile (Figure 2D, n = 14/group, LFD, 192.4 ± 29.48 s; HFD, 317.4 ± 32.75 s, p < 0.01), spent less time swimming (Figure 2E, n = 14/group, LFD, 339.2 ± 25.45 s; HFD, 153.3 ± 32.67 s, p < 0.001), and covered less distance, than the LFD group (Figure 2F, n = 14/group, LFD, 42.55 ± 5.22 cm; HFD, 33.6 ± 4.65 cm, p > 0.05) suggesting behavioral despair.

### 5.3 Thirty days of HFD does not alter anxiety-like behavior

**Figure 3:**
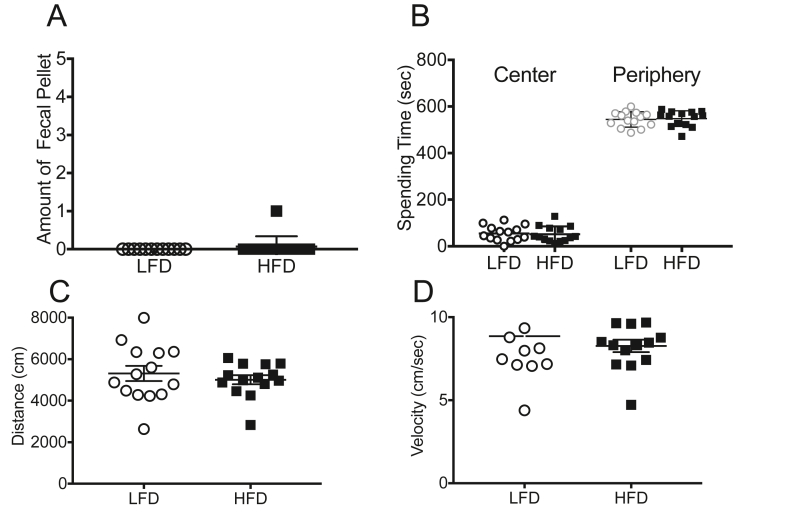
**Thirty days of HFD does not alter anxiety-like behavior.** After 30 days on HFD or LFD no differences were observed in anxiety-like behavior or motor capabilities in the open field test as assessed by (A) fecal pellet count, (B) time spent in the center and periphery of the area, (C) distance travelled, and (D) velocity. Data represented as mean ± standard error of the mean; n = 14/group.

The open field test measures anxiety-like behavior using a set of parameters including: time spent in center of the arena, distance, and velocity. Anxious animals tend to spend less time in the center. No differences were found between the LFD and HFD in fecal pellet counts (an indirect indicator of anxiety-like behavior) on day 30 in the open field (Figure 3A, n = 14/group). Consistent with the lack of difference in the fecal pellet counts, animals from both groups spent a similar amount of time in the center of the arena (Figure 3B, n = 14/group, LFD: 49.4 ± 8.51 s vs HFD 42.5 ± 7.10 s, p > 0.05) and in the periphery (Figure 3B, n = 14/group, LFD: 544.40 ± 8.81 s vs HFD 548.00 ± 8.97 s, p > 0.05) suggesting that the HFD did not alter anxiety-like behavior. To check for the presence of locomotor differences, distance and velocity were also assessed. No differences were found in either distance traveled (Figure 3C, n = 14/group, LFD, 5727 ± 353.50 cm v HFD, 5256 ± 154.1 cm, p > 0.05) or velocity (Figure 3D, n = 14/group, LFD, 8.5 ± 0.60 cm/s vs HFD, 8.3 ± 0.37 cm/s, p > 0.05) suggesting that both groups showed similar locomotion.

### 5.4 Rodents fed with HFD have greater colonic damage

**Figure 4:**
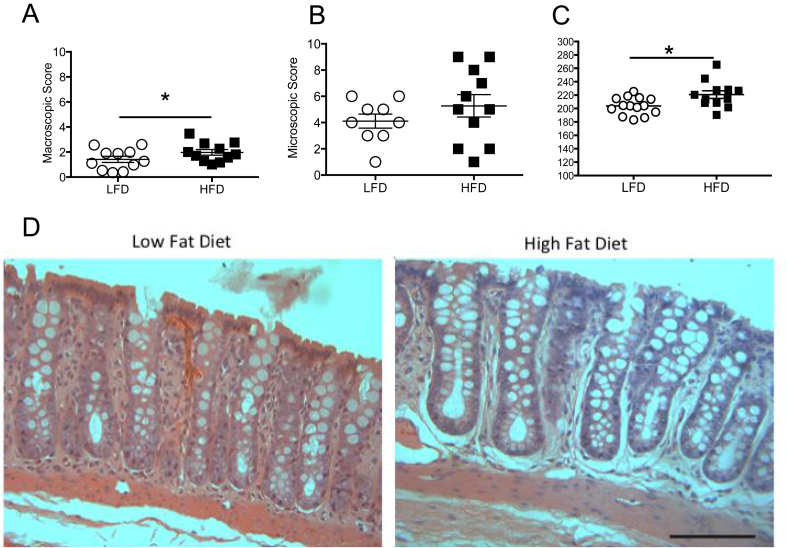
**Rodents fed with HFD have greater colonic damage**. Distal colon from HFD animals had (A) increased macroscopic damage (n = 11/group), (B) a trend towards increased microscopic damage (LFD, n = 9; HFD, n = 11), and (C) longer crypt length (LFD, n = 14; HFD, n = 12). (D) Representative colon tissue sections from animals fed with LFD or HFD. Data are shown as mean ± standard error of the mean. ∗p < 0.05, ∗∗p < 0.01. Scale bar = 100 μm.

After behavioral analysis, both groups were sacrificed and their colons were evaluated. We found that rodents in the HFD group had significantly greater macroscopic damage in the colon (Figure 4A, n = 11/group, LFD, 1.4 ± 0.25, HFD 1.9 ± 0.22, p < 0.01). Although there was a trend towards increased microscopic damage with more loss of crypt architecture, this did not reach significance (Figure 4B, LFD (n = 9), 4.1 ± 0.53, HFD (n = 11) 5.3 ± 0.85, p > 0.05). Interestingly, we observed that the average colonic crypt length of the HFD group was longer (Figure 4C, LFD (n = 14), 203.9 ± 3.44, HFD (n = 12), 220.8 ± 5.75, p < 0.05) than that found in the LFD group which suggests a possible hyperplasia and inflammation represented in photos from both groups (Figure 4D).

### 5.5 HFD rats have increased Bacteroides and decreased expression of tight junction proteins in the distal colon

**Figure 5:**
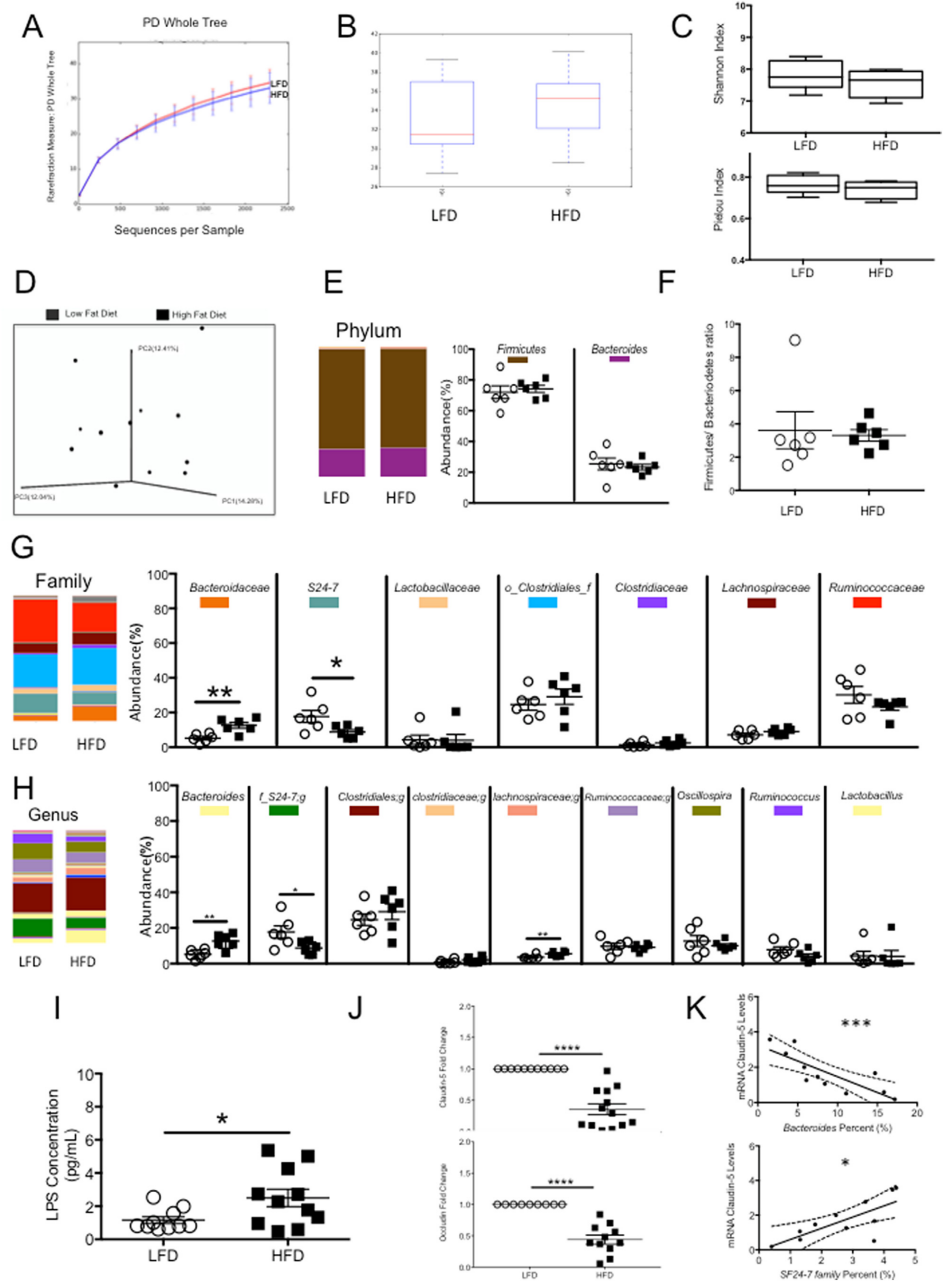
**HFD rats have increased *Bacteroide*s and decreased expression of tight junction proteins in the distal colon.** Alpha diversity shows no difference between HFD and LFD group (A) in PD Whole Tree, (B) Chao1 box plot, (C) Shannon index & Pielou index (p > 0.05; n = 6/group). (D) However, differences in beta diversity were found between the LFD and HFD animals (p < 0.05; n = 6/group). (G& H) Bacterial abundance was significantly different at both family and genus level, however (E&F) Firmicutes/Bacteroidetes abundance and ratio is similar in both groups (n = 6/group). (G & H) *Bacteroides* was significantly increased in HFD whereas *S24-7* family and genus levels were significantly decreased. (I) LPS levels in the blood were higher in the HFD group compared with the LFD group (LFD, n = 10; HFD, n = 11). (J) Analysis of tight junction proteins revealed lower mRNA expression of claudin-5 (LFD, n = 11; HFD, n = 13) and occludin in the HFD compared with LFD (LFD, n = 10; HFD, n = 11). (K) Significant correlations were found between % *Bacteroides* vs Claudin-5 mRNA, and % *S24-7* vs Claudin-5 mRNA. Data are represented as mean ± standard error of the mean (n = 11/group). P values ∗p < 0.05, ∗∗p < 0.01, and ∗∗∗p < 0.001.

After sacrifice, the fecal gut microbiome was analyzed for differences in alpha diversity, beta diversity, and taxonomy at phylum, family, and genus level (n = 6 in LFD group and n = 6 in HFD group). Alpha diversity showed no significant difference between the phylogenetic diversity whole tree (Figure 5A, n = 6/group; LFD, 31.41 ± 6.04, HFD 30.81 ± 7.15, p > 0.05), Chao1 Box Plots (Figure 5B, n = 6/group; LFD, 1263 ± 384.47, HFD1222.7 ± 459.59, p > 0.05), Shannon Index (Figure 5C, n = 6/group; LFD, 7.80 ± 0.19, HFD7.64 ± 0.17, p > 0.05), and Pielou Index (Figure 5C, n = 6/group; LFD, 0.76 ± 0.02, HFD0.74 ± 0.02, p > 0.05). The ANOSIM test showed however that the diet significantly contributed to the variability between the groups (p < 0.05, Figure 5D), thus contributing to the differences between the microbiomes of the HFD and LFD groups. At the phylum level, *Firmicutes*, *Bacteroidetes and Proteobacteria* were predominantly found with no significant differences between the two groups (*Firmicutes*-LFD, 71.98% ± 4.10; HFD, 74.13% ± 2.29, p > 0.05; *Bacteroidetes-LFD, 25.35%* ± 3.86, HFD, 23.30% ± 1.80, p > 0.05; Proteobacteria- LFD, 0.48% ± 0.14; HFD, 1.96% ± 1.15, p > 0.05 (not shown); Figure 5E; n = 6 in LFD group and n = 6 in HFD group). At the family level, *Porphyromonadaceae* LFD, 0.80% ± 0.25; HFD, 0.88% ± 0.28, p > 0.05; (not shown**)**; n = 6 in LFD group and n = 6 in HFD group), *Prevotellaceae* (LFD, 1.13% ± 0.37; HFD, 0.38% ± 0.11, p > 0.05;**(**not shown**)**; n = 6 in LFD group and n = 6 in HFD group)*, Lactobacillaceae* (LFD, 4.27% ± 2.67; HFD, 4.07% ± 3.32, p > 0.05; Figure 5G; n = 6 in LFD group and n = 6 in HFD group)*, Clostridiales* (LFD, 24.62% ± 3.20; HFD 29.12% ± 4.44, p > 0.05; Figure 5G; n = 6 in LFD group and n = 6 in HFD group), *Clostridiaceae* (LFD, 1.37% ± 0.57; HFD 2.48% ± 0.72, p > 0.05; Figure 5G; n = 6 in LFD group and n = 6 in HFD group)*, Lachnospiraceae* (LFD, 7.133% ± 1.00; HFD, 9.03% ± 0.76, p > 0.05; Figure 5G; n = 6 in LFD group and n = 6 in HFD group), and *Ruminococcaceae* (LFD, 30.23% ± 4.83; HFD, 23.32% ± 2.02, p > 0.05; Figure 5G; n = 6 in LFD group and n = 6 in HFD group) were found with no differences in composition between the groups; however, *Bacteroidaceae* were increased in HFD group compared with LFD group (Figure 5G; n = 6/group, LFD, 5.2% ± 1.01, HFD 12.6% ± 1.67, p < 0.01). Interestingly, we observed that the average whereas *S24-7* were decreased in the HFD compared to LFD (n = 6/group, LFD, 4.27% ± 2.66, HFD 4.1% ± 3.32, p < 0.05) and *Streptococcacea* (LFD, 1.20% ± 0.43; HFD 0.08% ± 0.047, p < 0.05; **not shown**; n = 6 in LFD group and n = 6 in HFD group) in a low percent of abundance. At the genus level, for *Lactobacillus* (LFD, 4.27% ± 2.66; HFD, 4.072% ± 3.32, p > 0.05; Figure 5H; n = 6 in LFD group and n = 6 in HFD group)*, genus of Clostridiales* (LFD, 424.62% ± 3.21; HFD, 291.12% ± 4.43, p > 0.05; Figure 5H; n = 6 in LFD group and n = 6 in HFD group)*, genus of Clostridiaceae* (LFD, 0.97% ± 0.45; HFD, 2.03% ± 0.62, p > 0.05; Figure 5H; n = 6 in LFD group and n = 6 in HFD group)*, genus of Ruminococcaceae* (LFD, 9.73% ± 1.74; HFD, 9.11% ± 0.87, p > 0.05; Figure 5H; n = 6 in LFD group and n = 6 in HFD group)*; Oscillospira* (LFD, 012.65% ± 3.11; HFD, 10.12% ± 1.04, p > 0.05; Figure 5H; n = 6 in LFD group and n = 6 in HFD group), and *Ruminococcus* (LFD, 7.8% ± 1.61; HFD, 4.05% ± 1.33, p > 0.05; Figure 5H; n = 6 in LFD group and n = 6 in HFD group) no differences were found in bacteria composition between the diets (Figure 5H; n = 6 in LFD group and n = 6 in HFD group; p > 0.5) nor in the F/B Ratio between the groups (Figure 5F n = 6/group, LFD, 3.6 ± 1.11, 3.3 ± 0.34, p > 0.05). However, the HFD group showed a significant increase in percent of *Bacteroides* (Figure 5H, n = 6/group, LFD, 5.2% ± 1.05, HFD 12.63% ± 1.67, p < 0.01) and in *Lachnospiraceae* (LFD, 3.65% ± 0.53; HFD, 5.57% ± 0.61, p < 0.05; Figure 5H; n = 6 in LFD group and n = 6 in HFD group). Interestingly, we observed that the average *S24-7* bacteria decreases in HFD compared with in LFD group (Figure 5H, n = 6/group, LFD, 17.2% ± 3.46, HFD 8.75% ± 1.38, p < 0.05). LPS levels in the blood were higher in HFD compared to LFD group (Figure 5I, n = 10 LFD 1.1 pg/mL ± 3.46; n = 11, HFD 2.5 pg/mL ± 0.58, p < 0.05). We also examined the expression of the tight junction proteins claudin-5 and occludin in colonic tissue. We found reduced expression of claudin-5 (Figure 5J, n = 11 LFD 1.0 ± 0; n = 13, HFD.3592 ± 0.08 p < 0.0001) and occludin (Figure 5J, n = 9 LFD, 1.0 ± 0; n = 13, HFD 0.4 ± 0.07 p < 0.0001) in the colon of animals in the HFD group (Figure 5J), which may suggest a leaky gut. To determine if the changes in microbiome were related to the reduced expression of tight junction genes, Spearman correlations were performed between *Bacteroides* and *S24-7* abundance and tight junction expression. *Bacteroides* abundance was negatively correlated with claudin-5 expression (−0.87, p < 0.001, Figure 5K; n = 5–6 per group), while *S24-7* abundance positively correlated with claudin-5 expression (0.71, p < 0.05).

### 5.6 HFD rats exhibit alterations in astrocyte morphology with increased microglia percent area & intensity in the CA1, and decreased Phospho-mTOR in the cingulate cortex

**Figure 6:**
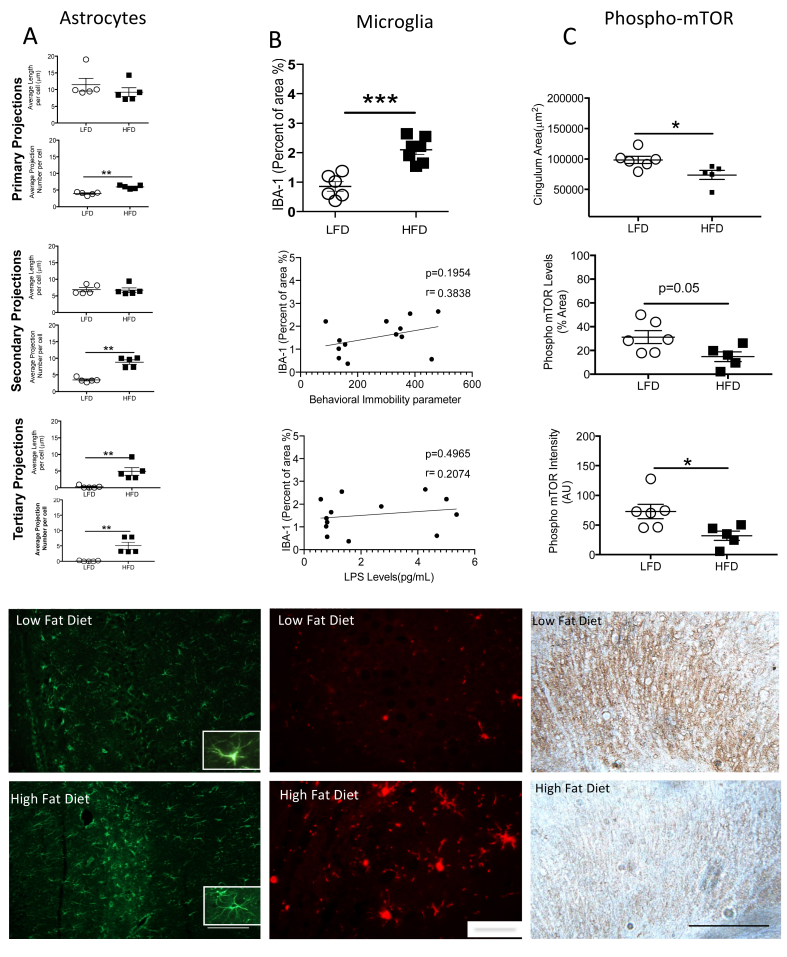
HFD rats exhibit higher astrocytic branching in projections, higher percent area of microglia and lower expression of Phospho-mTOR in the brain. (A) HFD group show increased hippocampal astrocytic projection length in the secondary and the tertiary projections respectively. However, HFD show a higher number of projections from the primary to the tertiary projections compared with the LFD n = 5/group. (B) Hippocampal IBA-1 staining, show higher increase in percent area and intensity in the HFD group (n = 7) compared with the LFD group (n = 6). On the contrary, association of the immobile parameter and IBA-1 percent of area and LPS concentration vs IBA-1 show a trend but did not reach significance. (C) The area and intensity of phospho-mTOR levels were decreased in the HFD (n = 5) group compared with the LFD group (n = 6). In addition, the area of the cingulum in the dorsal hippocampus showed a significant decrease in HFD group compared with the control. Data are represented as mean ± standard error of the mean. ∗p < 0.05, ∗∗p < 0.01, and ∗∗∗p < 0.001.

Brain sections were removed to assess the alterations of the astrocytes and the microglia in the CA1. Glial Fibrillary Acid Protein (GFAP), primary astrocytic projection length, showed no differences between the groups (Figure 6A, LFD (n = 5), 11.5 μm ± 1.88, HFD (n = 5), 9.2 μm ± 1.32, p > 0.05), however, differences in primary astrocytic projection number between the groups was increased **(**LFD (n = 5), 3.9 ± 0.21, HFD (n = 5), 5.9 ± 0.24, p < 0.01). HFD and LFD groups showed no difference in secondary astrocytic projections length (LFD (n = 5), 6.9 μm ± 0.61, HFD (n = 5), 6.72 μm ± 0.69, p > 0.05), but HFD showed higher secondary astrocytic projections number compared with the LFD group (LFD (n = 5), 3.49 ± 0.29, HFD (n = 5), 8.9 ± 0.65, p < 0.01). HFD group exhibits higher tertiary astrocytic projections length (LFD (n = 5), 0.23 μm ± 0.17, HFD (n = 5), 4.88 μm ± 1.15, p < 0.01) and tertiary astrocytic projections number (LFD (n = 5), 0.11 ± 0.06, HFD (n = 5), 5.072 ± 1.15, p < 0.01). In addition, the microglia marker (IBA-1) (Figure 6B) showed higher percent area (LFD (n = 6), 0.86 ± 0.16, HFD (n = 7), 2.10 ± 0.16, p < 0.01) in the HFD rats compared with the control, on the contrary correlations between IBA-1 vs. immobility (p > 0.05, n = 13), and IBA-1 vs LPS concentration in the blood (p > 0.05, n = 13) did not reach significance. Finally (Figure 6C), HFD showed decreased cingulum cortex area stained against Phospho-mTOR (LFD (n = 6), 98501μm^2^ ± 5936, HFD (n = 5), 73742 μm^2^ ± 7523, p < 0.05), Phospho-mTOR percent area (LFD (n = 6), 31.2% ± 5.42, HFD (n = 5), 14.76% ± 4.12, p = 0.05), and Phospho-mTOR intensity (LFD (n = 6), 72.8 ± 12.21, HFD (n = 5), 32.0% ± 7.98, p < 0.05) compared with the LFD group. Scale bars show 100 μm for GFAP, IBA-1, and Phospho-mTOR representative photos.

### 5.7 Leptin levels were significantly higher in rodents with HFD

Markers of inflammation such as interleukin-1 beta (IL-1β), tumor necrosis alpha (TNF-α), monocyte chemoattractant protein-1 (MCP-1 or CCl-2), and interleukin 6 (IL-6), and markers related to obesity such as leptin and insulin were measured systemically in the blood. As shown in Table 2, no differences were found in inflammatory cytokines; however, there were significantly higher levels of leptin in the HFD group compared with the LFD group. Additionally, HFD animals had significantly higher LPS levels (p < 0.05; Figure 5I) (see Table 1).

**Table 1:** Formulation of the Low-Fat Diet and High-Fat Diet given to male Sprague-Dawley rats for 30 days^a^ Ingredients calculated to 1000 g of diet based on the Information from Research Diets, Inc. (https://www.researchdiets.com).

Component	Ingredient	Low-Fat diet (D12450J) (g/kg of diet)^a^	High-Fat diet (D12492) (g/kg of diet)^a^
Protein	Casein, Lactic, 30 Mesh	189.50 g	258.25 g
	Cystine, L	2.84 g	3.88 g
	Starch, Corn	479.79 g	0 g
Carbohydrate	Lodex	118.48 g	161.53 g
	Sucrose, Fine Granulated	69.00 g	94.08 g
	Solka Floc, FCC200	47.39 g	64.61 g
Fat	Soybean Oil, USP	23.70 g	32.31 g
	Lard	18.96 g	316.60 g
Mineral Mix	S10026B	47.39 g	64.61 g
Vitamin	Choline Bitartrate	1.90 g	2.58 g
	V10001C (Vitamin mix)	0.95 g	1.29 g
Dye	Dye, Yellow FD&C #5, Alum. Lake 35–42%	0.04 g	0 g
	Dye, Blue FD&C #1, Alum. 35–42%	0.01 g	0.06 g
Total		1000 g	1000 g
			
Caloric Information			
	Protein	20 % kcal	20 % kcal
	Fat	10 % kcal	60 % kcal
	Carbohydrate	70 % kcal	20 % kcal
	Energy Density	3.82 kcal/g	5.21 kcal/g

**Table 2:** **Protein Array of inflammatory cytokines and insulin in the serum.** Two-month-old male Sprague Dawley on LFD or HFD show no significant differences in inflammatory cytokine levels (IL-1β, IL-6, TNFα, CCL2, Insulin). Higher levels of leptin were found in the rats fed with HFD compared with those on LFD. Data represented as mean ± standard error of the mean. N = 6/group. Statistics used student t-test. ∗p < 0.05.

Blood Marker	LFD (pg/mL)	HFD (pg/mL)	p value
IL-1β	0.91 ± 0.35	0.57 ± 0.01	>0.05
IL-6	Below detection	Below detection	>0.05
TNF-α	0.15 ± 0.09	0.09 ± 0.01	>0.05
CCL-2	76.59 ± 10.49	86.77 ± 11.94	>0.05
Insulin	1998 ± 448.10	2025 ± 784.00	>0.05
Leptin	**1408 ± 193.00**	**2078 ± 174.00**	**<0.05**

## 6 Discussion

In this study, we used an animal model to better understand the early changes between the gut and the peripheral system even before obesity is apparent. Our results show that Sprague Dawley rats fed an HFD for only one month demonstrated behavioral despair even without excessive weight gain. Further, consumption of the HFD for this short time period altered beta diversity and bacterial abundance in the lower taxonomic levels, increasing *Bacteroides* abundance, which correlated with decreased expression of tight junction proteins and LPS in the blood suggesting a leaky gut. Even more, the metabolites present in the blood such as leptin, free fatty acid, and LPS produce alterations in the brain that may be associated to behavior despair.

The gut microflora helps to maintain the physiology and histology of the colon (Belkaid and Hand., 2014), and can be affected by many factors including dietary components (David et al., 2014; Wu et al., 1998). In the present study we found significant differences in the beta diversity of the fecal microflora between an HFD and LFD even without obesity, however we did not see differences in alpha diversity. In addition, we observed the taxonomy at family and genus level and found that the percentage of *Bacteroide* and *Bacteroides* was significantly higher in the animals fed with HFD, which echoes other reports of HFD impact (Wu et al., 1998; Schnorr et al., 2014). In addition, the S24-7 family was significantly lower in rats fed with HFD. This bacterium is not well characterized (Salzman et al., 2002; Ormerod et al., 2016), but appears to be related to gut composition in rodents fed with low fat diet undergoing exercise (Evans et al., 2014) and was observed during remission in a colitis mouse model (Rooks et al., 2014). *Bacteroides* is an obligate anaerobic bacterium that provides beneficial properties to the gut including survival under different environments and can digest plant and host polysaccharides (Almeida-Suhett et al., 2017; Belkaid and Hand, 2014; Gecici et al., 2005; Methé et al., 2012). Different species of *Bacteroides* have been studied for their polysaccharide utilization loci (PUL) since the bacteria have the capacity to recognize, translocate, hydrolyze, and regulate polysaccharide genes (Methé et al., 2012; Hooper et al., 2001; Dutheil et al., 2016; Hsiao et al., 2013). However, in diets with low fiber, such as a high fat diet, *Bacteroides* can digest the glycans present in the gut (Sonnenburg et al., 2005). This can be detrimental since O-glycans are a major component of mucin 2, secreted by goblet cells, which normally comprise a mucus barrier separating the gut microflora from the epithelium of the host. A decreased mucus barrier is associated with epithelial cell damage and diets lower in fiber (Johansson et al., 2008). Such diets have been associated with inflammatory bowel diseases (IBD) or cancer (Cameron and Sperandio, 2015), while those high in fiber may reduce the risk of developing IBD (Owczarek et al., 2016). In the present study the HFD contained no starch and this perhaps might be associated with a decreased layer of mucus, contributing to the inflammation observed although this was not specifically measured.

We found that a short-term HFD significantly increased macroscopic damage in the colon and altered crypt length, suggesting presence of inflammation (Erben et al., 2014). Indeed, the expression of the tight junction proteins, Claudin-5 and Occludin, were significantly decreased in the colon. Such a decreased expression has previously been associated with a leaky gut, and linked to an HFD in humans and rodent models (Barmeyer et al., 2017; Alhasson et al., 2017; Camilleri et al., 2012; Jakobsson et al., 2015; Rose et al., 2012; Sánchez-Villegas et al., 2012; Rahner et al., 2001). Interestingly, the increased presence of *Bacteroides* species, as found in our study, has also been associated with changes in intestinal permeability with an HFD (Cani et al., 2008; Hooper et al., 2001; David et al., 2014). We observed a positive correlation between Claudin-5 and S24-7, whereas a negative correlation was found with *Bacteroides*. In line with our results, various colonic conditions such as acute colitis and Crohn's disease have also found decreased expression of claudin-5 associated with increased *Bacteroides* abundance (Mennigen et al., 2009; Rosen et al., 2011). In general, *Bacteroides* has been shown to negatively impact patients with ulcerative colitis and influence symptoms found in patients with inflammatory bowel diseases (Kuwahara et al., 2004; Setoyama et al., 2003; Matsuda et al., 2000; Hansen et al., 2012; Hudcovic et al., 2009). *Bacteroides* can produce a toxin called fragilysin, which may disrupt the epithelial paracellular barrier (Obiso et al., 1997) through proteolytic degradation of the extracellular domain on E-cadherin on intestinal cells, resulting in junction disassembly (Wu et al., 1998, 2011). The lower expression of tight junctions we observed might therefore contribute to translocation of bacterial products, such as LPS, and increased gut permeability. As gut permeability increases, LPS can enter through the tight junctions and then be transported inside chylomicrons to reach the circulation to produce low-grade inflammation. In humans an HFD was shown to increase this transport (Ghoshal et al., 2009). LPS can then activate toll like receptors, specifically toll like receptor 4, which are present in the colonic epithelium and immune cells (Abreu et al., 2010, Yiu et al., 2017; Medzhitov, 2007) and consequently stimulate the NFKB pathway increasing inflammatory cytokines (Cook et al., 2004; Billack, 2006). Interestingly, *Bacteriodes* also has the ability to change the polysaccharide surface mediated by an invertase gene called mpi, which has the capacity of immune evasion (Coyne et al., 2003). In addition, LPS also increases white adipose tissue and inflammatory markers affecting the brain behavior (André et al., 2014; Zhao et al., 2019).

We observed that although our HFD group showed no significant increase in absolute weight during the intervention, the animals had increased epididymal fat pads, and increased leptin in the blood suggesting metabolic changes. A significant increase in percent weight gain was observed compared to those animals fed LFD but the difference did not reach the commonly accepted definition of obesity (Hariri and Thibault, 2020). The presence of increased epididymal fat pads following a short-term (2 week) HFD in rats has previously been observed (Li et al., 2002), and it is known that high levels of LPS in the blood may produce blood brain barrier (BBB) permeability resulting in increased leptin and inflammatory cytokines, such as interleukin 1β (IL-1β) and interleukin 6 (IL-6) (Lago et al., 2007; Banks et al., 2015; Nishioku et al., 2009). This BBB permeability may permit entry of products producing activation of toll like receptors in the brain in different cell types such as microglia (Liu et al., 2014; Chakravarty and Herkenham, 2005), causing brain inflammation that then leads to changes in behavior. It has been shown that LPS, IL-1β, and IL-6 injected peripherally can produce depressive-like behavior (Anforth et al., 1998; Walker et al., 2013; Wang et al., 2011), and free fatty acids (FFA) can activate the microglia in vitro in a TLR-4 dependent manner (Wang et al., 2012; Lee et al., 2001, 2011). Interestingly, decreased adiposity and inflammation have been shown in TLR-4 knockout mice (Saberi et al., 2009; Tsumuko et al., 2007) and behavioral despair has been observed when TLR-4 is inhibited or knocked out (Zhang et al., 2020).

In this study, we used an HFD which contained lard as the major source of fat. This diet had roughly equivalent levels of saturated fat (SFA; 32%), monounsaturated fat (MUFA; 36%), and polyunsaturated fat (PUFA; 32%). Several studies show the importance of MUFA and PUFA on the brain and behavior (Levant, 2013; Hryhorczuk et al., 2016) where an imbalance (higher ratio of omega-6: omega-3) is associated with depressive-like behavior (du Bois et al., 2006; Tang et al., 2016). Our diet is high in SFA, specifically palmitic acid, that has been associated with immobility in the forced swim test (Sharma and Fulton, 2013; Kaczmarczyk et al., 2013). Further, in humans, an association has been found between systemic palmitic acid levels and symptoms of depression (Tsuboi et al., 2013). Our data is thus comparable with other studies showing that consumption of a diet high in saturated fat increases FFA resulting in adiposity even over a short time period (Lee et al., 2001, 2011; Horton et al., 1995; Phillips et al., 2012; Nguyen et al., 2007). In our short timeframe, the HFD group had significantly increased immobility with decreased swimming time and less distance traveled in the forced swim test, supporting the hypothesis that an HFD can lead to behavioral despair. These data correlate with other studies, which found depressive-like behavior in rodents consuming an HFD over a longer time frame of 10 weeks (Abildgaard et al., 2011; Sharma and Fulton, 2013). In our study we found no anxiety-like behavior in the HFD group as measured by the open field test. These findings corroborate those found previously in rodents fed with HFD for 6 weeks, indicating that the depressive-like syndrome is not due to sickness behavior nor anxiety (Gainey et al., 2016). On the contrary, when the rats were fed with HFD for a longer time period, more than 15–17 weeks (Dutheil et al., 2016), it is notable that these authors did see an increase of anxiety-like behavior in the open field test, again pointing to temporal effects of the diet on behavior. It is possible then that the data discrepancies can be due to differences in rat strains, diet and performed tests. However, it is important to recall the importance of neuroinflammation and immune cells in the brain.

Immune cells can be found in the brain especially in the hippocampus. In our research, we demonstrated that HFD caused a reactive state in astrocytes by increasing the number of primary, secondary, and tertiary projections per cell, suggesting hyperactivity and increased microglia levels in the hippocampus, CA-1. We also found decreased phospho-mTOR in the cingulate cortex. The hippocampus has been studied for its role in memory formation and depressive like syndrome, and under normal conditions sends information to the subiculum and to the entorhinal cortex (van Groen et al., 2014). However, under neuro inflammation conditions, the mTOR signaling is inhibited (Dutheil et al., 2016, Huang and Reichardt, 2001; Li et al., 2010, Sengupta et al., 2010) resulting in a decrease of the derived neurotropic factor (BDNF) levels affecting synaptogenesis. In addition, short- and long-term HFD exposure in animal models has been associated with increased dysregulation of astrocytes and microglia (Sofroniew and Vinters, 2010; Calvo-Ochoa et al., 2014; Rorato et al., 2022). The TLR4 receptor in the brain is known to express pro-inflammatory cytokines (Hanke el at, 2011) and inflammation can decrease phospho-mTOR, which is important for the synthesis of BDNF (Dutheil et al., 2016). In response to LPS microglial cell staining showed increased percent area for Iba-1 positive cells, denoting the possibility of a neuroinflammatory process in the CA-1, which is a key structure related to an enhanced response to antidepressant treatments (Rolls et al., 2018; Taylor et al., 2014), and further resolution of depressive symptoms. As well, mTOR signaling has been confirmed as an important modulator of protein synthesis including synaptic protein synthesis, which is deregulated in behaviors such as major depressive disorder or depression (Ignácio et al., 2016; Abelaria et al., 2014). We demonstrate here that short-term HFD exposure reduces the cingulum cortex area stained against Phospho-mTOR, which agrees with what has already been published, where exposure to HFD reduces mTOR staining and mTOR mRNA expression in brain tissue (Oh et al., 2013; Dasuri et al., 2016; Arnold et al., 2014). In addition, we found a decrease in cingulum area which may be associated to human studies where depressed patients have lower fractional anisotropy (Won E et al., 2016). Interesting, lesions in the cingulum are associated to depression (Taylor et al., 2014). In sum, these data suggest that the presence of immune cells such as astrocytes and microglia may affect the hippocampal connectivity by affecting the BDNF expression inhibiting mTOR signaling.

**Figure 7:**
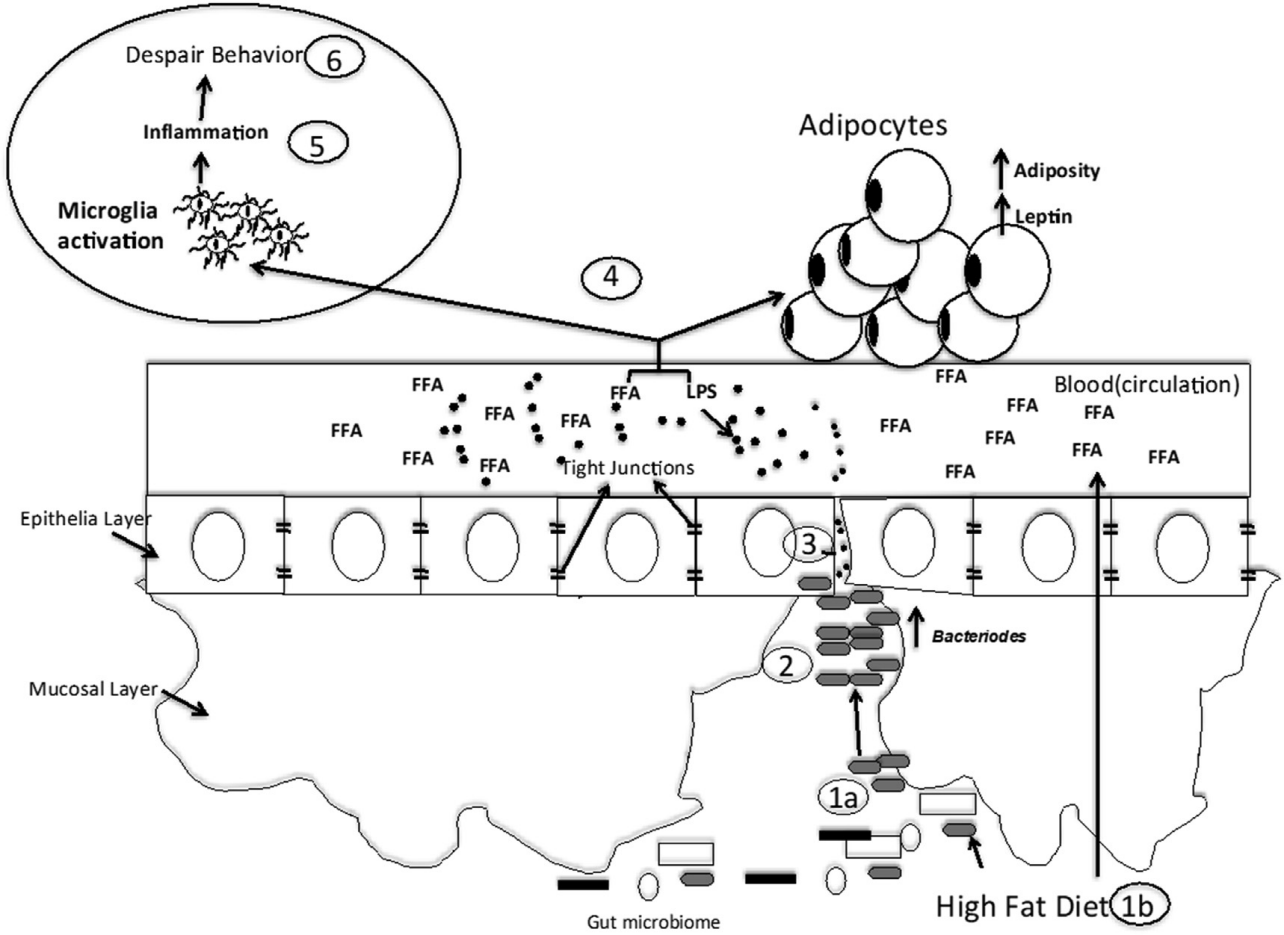
**HFD conceptual model.** (1a) An HFD (unsaturated fat) produces an increase in *Bacteroides* abundance and also the gut increasing Free Fatty Acids (FFA) in the blood absorbs (1b). (2) *Bacteroides* digest the mucins in the host when low levels of starch are present (as found in the HFD). (3) Toxins from the *Bacteroides* decrease tight junction mRNA levels promoting the entry of LPS (represented by black dots) to the peripheral system. (4) Circulating LPS and FFA increases adiposity and leptin levels, and possibly activates microglia in a TLR4 dependent manner. (5) TLR4 pathway produces inflammation in the brain by cytokines. (6) Brain inflammation produces behavioral despair.

Based on our findings and the literature, we accept our hypothesis but acknowledge limitations (see the limitations section) and propose a conceptual model for the impact of a short-term high-fat diet on behavior taking into consideration our findings and the literature (Figure 7): consumption of a high fat diet over even a short period of time will be associated with increased abundance of *Bacteroides* and increased circulating FFA in the blood (van Dijk et al., 2009; Rosqvist et al., 2014). In the colon, this low fiber diet promotes digestion of the mucin in the host (Benjdia et al., 2011; Martens et al., 2009; Turroni et al., 2010) resulting in decreased expression of tight junction proteins such as occludin and claudin-5 and entrance of bacterial products such as LPS into the circulation. Circulating LPS and FFA may activate TLR-4 pathways in adipose tissue resulting in increased leptin levels. In addition, circulating LPS and FFA may activate immune cells in the brain such as microglia (Krinos et al., 2001; Chatzidaki-livanis et al., 2010). This results in increased brain inflammation and promotes behavioral despair (Millett et al., 2019; O'Connor et al., 2009).

### 6.1 Limitations of the study

Our study has several limitations that may restrict some of the conclusions that we are able to draw since some of the data is correlative. We only analyzed the microbiome on day 30 at sacrifice, whereas collection of fecal pellets for analysis at day 0 and Day 15 would have helped us to better understand the microbiome shifts during the intervention. Only one behavioral test (forced swim) was used to measure ‘despair’ and the results of this test could be interpreted not only as the animals giving up trying to escape from the situation, but also as a failure to cope. Ideally, in follow up experiments to define the behavioral changes as being equivalent to a depressive-like behavior, additional tests such as social defeat (social aversion), the sucrose preference test (anhedonia) and nest building (apathy) could be used (Planchez et al., 2019). Finally, we did not assess the expression of the possible responsible receptors, such as TLR4, in the colon, feces or the brain, nor performed inhibition of TLR4 and inflammatory cell in the colon. Follow up investigations might directly measure gut permeability, TLR4, mp1 or endotoxin to identify which parameter LPS uses to enter to the blood stream, and examine in more depth the role of the S24-7 genus. In future studies such further investigations may shed additional light on the various mechanistic pathways that may be involved in generating the behavioral changes.

## Declarations

### Author contribution statement

Gladys Chompre: Conceived and designed the experiments; Performed the experiments; Analyzed and interpreted the data; Contributed reagents, materials; analysis tools or data; Wrote the paper.

Lubriel Sambolin; Myrella L Cruz; Yarelis Rodriguez: Performed the experiments; Analyzed and interpreted the data, analysis tools or data; Wrote the paper.

Rafael Sanchez: Performed the experiments; Analyzed and interpreted the data analysis tools or data; Contributed reagents, materials, analysis tools or data; Wrote the paper.

Ronald E Rodriguez-Santiago: Analyzed and interpreted the data; Contributed reagents, materials, analysis tools or data; Wrote the paper.

Yasuhiro Yamamura: Conceived and designed the experiments; Analyzed and interpreted the data; Contributed reagents, materials,analysis tools or data; Wrote the paper.

Caroline B Appleyard: Conceived and designed the experiments; Analyzed and interpreted the data; Contributed reagents, materials, analysis tools or data; Wrote the paper.

### Funding statement

Associate Professor Gladys Chompre was supported by Foundation for the 10.13039/100000002National Institutes of Health [P20 GM103475].

Yasuhiro Yamamura was supported by AIDS Research Infrastructure Program and Behavioral Core [RR003050/MD007579].

Caroline B Appleyard was supported by the 10.13039/100000002National Institutes of Health [2R25GM082406].

### Data availability statement

Data associated with this study has been deposited at SRA under the accession number PRJNA870914 [https://www.ncbi.nlm.nih.gov/sra/PRJNA870914].

### Declaration of interest’s statement

The authors declare no conflict of interest.

### Additional information

No additional information is available for this paper.

## Appendix A Supplementary data

SupplementalDataFatCompositionFinal.docx SupplementalDataFatCompositionFinal.docx
