# Supplementary material for: A one month high fat diet disrupts the gut microbiome and integrity of the colon inducing adiposity and behavioral despair in male Sprague Dawley rats
Source: Heliyon. 2022 Oct 27;8(11):e11194. doi: 10.1016/j.heliyon.2022.e11194 (PMC9663868; doi:10.1016/j.heliyon.2022.e11194)
Supplement: SupplementalDataFatCompositionFinal.docx [file mmc1.docx]

|  | **Ingredient** | grams |  | grams |  |
| --- | --- | --- | --- | --- | --- |
|  | Lard | 19.98 |  | 316.6 |  |
|  | Soybean Oil | 23.7 |  | 32.31 |  |
|  | **Total Fat** | **43.68** |  | **348.91** |  |
|  |  |  |  |  |  |
|  | | LFD (12450J) | | HFD (D12492) | |
|  | | grams | % of total fat | grams | % of total fat |
|  | C10, Capric | 0.00 | 0.00 | 0.13 | 0.04 |
|  | C12, Lauric | 0.00 | 0.00 | 0.26 | 0.07 |
|  | C14, Myristic | 0.29 | 0.67 | 3.62 | 1.04 |
| SFA | C15 | 0.00 | 0.00 | 0.26 | 0.07 |
|  | C16, Palmitic | 6.21 | 14.22 | 64.48 | 18.48 |
|  | C17 | 0.10 | 0.22 | 1.16 | 0.33 |
|  | C18, Stearic | 3.01 | 6.89 | 34.76 | 9.96 |
|  | C20, Arachidic | 0.10 | 0.22 | 0.65 | 0.19 |
|  | C22, Behenic | 0.10 | 0.22 | 0.13 | 0.04 |
|  |  |  |  |  |  |
|  | C16:1, Palmitoleic, n-9 | 0.29 | 0.67 | 4.39 | 1.26 |
| MUFA | C18:1, Oleic, n-9 | 11.94 | 27.33 | 111.52 | 31.96 |
|  | C20:1 | 0.19 | 0.44 | 2.07 | 0.59 |
|  |  |  |  |  |  |
|  | C18:2, Linoleic | 17.28 | 39.56 | 93.95 | 26.93 |
|  | C20:2 | 0.19 | 0.44 | 2.58 | 0.74 |
|  | C18:3, Linolenic | 2.04 | 4.67 | 6.59 | 1.89 |
| PUFA | C20:3, n-6 | 0.00 | 0.00 | 0.39 | 0.11 |
|  | C20:4, Arachidonic, n-6 | 0.10 | 0.22 | 0.90 | 0.26 |
|  | C22:5,Docosapentaenoic, n-3 | 0.00 | 0.00 | 0.26 | 0.07 |
|  |  |  |  |  |  |

**Supplemental Table 1.** Fat composition (saturated fatty acid (SFA), monosaturated fatty acid (MUFA), and polysaturated fatty acid (PUFA) of the Low-Fat Diet and High-Fat Diet given to male Sprague-Dawley rats for 30 days

^a^ Fat Composition was modified using the calculated fat ingredient in table 1 and this data was provided by Research Diets, Inc. (https://www.researchdiets.com)
